# Sonazoid-Conjugated Natural Killer Cells for Tumor Therapy and Real-Time Visualization by Ultrasound Imaging

**DOI:** 10.3390/pharmaceutics13101689

**Published:** 2021-10-15

**Authors:** Hyeong-Woo Song, Han-Sol Lee, Seok-Jae Kim, Ho Yong Kim, You Hee Choi, Byungjeon Kang, Chang-Sei Kim, Jong-Oh Park, Eunpyo Choi

**Affiliations:** 1 Korea Institute of Medical Microrobotics, Gwangju 61011, Korea; shw@kimiro.re.kr (H.-W.S.); hansol3607@kimiro.re.kr (H.-S.L.); outcry9@kimiro.re.kr (S.-J.K.); kimhy16@kimiro.re.kr (H.Y.K.); angelgod305@kimiro.re.kr (Y.H.C.); bjkang8204@jnu.ac.kr (B.K.); ckim@jnu.ac.kr (C.-S.K.); 2 School of Mechanical Engineering, Chonnam National University, Gwangju, 61186, Korea; 3 College of AI Convergence, Chonnam National University, Gwangju 61186, Korea

**Keywords:** NK cells, microbubbles, solid tumors, ultrasound, imaging

## Abstract

Various cell therapy strategies, including chimeric antigen receptor-expressing T or natural killer (NK) cells and cell-mediated drug delivery, have been developed for tumor eradication. However, the efficiency of these strategies against solid tumors remains unclear. We hypothesized that real-time control and visualization of therapeutic cells, such as NK cells, would improve their therapeutic efficacy against solid tumors. In this study, we engineered Sonazoid microbubble-conjugated NK (NK_Sona) cells and demonstrated that they were detectable by ultrasound imaging in real-time and maintained their functions. The Sonazoid microbubbles on the cell membrane did not affect the cytotoxicity and viability of the NK cells in vitro. Additionally, the NK_Sona cells could be visualized by ultrasound imaging and inhibited tumor growth in vivo. Taken together, our findings demonstrate the feasibility of this new approach in the use of therapeutic cells, such as NK cells, against solid tumors.

## 1. Introduction

Natural killer (NK) cell therapy, one of several immune-based therapeutic strategies, is still under intense study for its use as a cancer treatment [1]. Some immune cell therapies, including chimeric antigen receptor (CAR)-expressing T-cell therapy or CAR-expressing NK cell therapy, have been successful against liquid tumors, such as lymphomas and leukemias [2,3,4]. However, despite numerous studies and attempts, the use of immune cell therapy for curing solid tumors has proven difficult owing to the inability of the immune cells to infiltrate the tumor. Specifically, effective tumor penetration by the immune cells is impeded by the tumor microenvironment [5], which contains immune cell inhibitory and regulatory factors and an extracellular matrix that work in concert to protect the tumor cells [6,7]. Consequently, diverse methods have been attempted to mitigate these problems to allow tumor penetration by the immune cells and their activation locally and specifically [8,9,10].

One such strategy—sonoporation—which involves the use of ultrasound and microbubbles to enhance target cell membrane permeability, has improved the delivery efficiency of drugs that are in the form of mRNAs, antibodies, and immune cells [11,12,13]. One study demonstrated that the solid tumor-penetrating effect of NK-92MI cells, which were co-treated with microbubbles, was enhanced by the use of focused ultrasound [14]. A major advantage of combined ultrasound and microbubble use is the ability to release the therapeutic drug locally, which leads to fewer side effects. Moreover, as a minimally invasive diagnostic method with a proven safety record, ultrasound has already been used clinically for the diagnosis and therapy of tumors.

While microbubble contrast agents have been used widely as a strategy for seeking tumors, many researchers are now studying their potential therapeutic application for enhancing the functions and delivery of drugs [15,16,17]. Various therapeutic agents, such as chemical drugs, mRNAs and antibodies, can be conjugated to or encapsulated in microbubbles owing to their intrinsic carrying capacity [11,15,18]. Aside from drugs, cells can also be coupled to microbubbles. For example, mesenchymal stem cell–microbubble conjugates, named StemBells, have been developed for the enhancement of cardiac function [19]. The microbubbles on the StemBells are able to recognize both CD90 on stem cells and ICAM-1 on endothelial cells, allowing the increased ultrasound-directed attachment of the conjugates to the infarct area compared with that of free stem cells.

We hypothesized that NK cells would maintain their viability and function even when conjugated with microbubbles. Herein, we describe an alternative model for cell therapy of solid tumors, based on the conjugation of microbubbles to the NK cell membrane, and real-time imaging of the therapeutic effect. Sonazoid, an approved microbubble contrast agent in several countries, and used particularly for the detection of liver tumors [20], was used to engineer the NK cell–microbubble complexes.

## 2. Materials and Methods

### 2.1. Cell Lines and Reagents

NK-92^®^ MI cells were purchased from the American Type Culture Collection (ATCC, Rockville, MD, USA) and were cultured in Minimum Essential Medium Alpha (Gibco) supplemented with 12.5% fetal bovine serum (FBS, Corning, New York, NY, USA), 12.5% horse serum (Corning), 0.2 mM inositol, 0.1 mM β-mercaptoethanol, and 0.02 mM folic acid. A549 cells were purchased from the Korean Cell Line Bank (Seoul, Korea) and cultured in RPMI 1640 medium (Corning) supplemented with 10% FBS (Corning). All of the cell lines were cultured at 37 °C in a humidified atmosphere containing 5% CO_2_.

### 2.2. Construction of Sonazoid-Conjugated Natural Killer Cells

The conjugation of Sonazoid (GE Healthcare, Chicago, IL, USA) to the membrane of NK cells was carried out using the protocols described in previous studies [19,21,22]. Sonazoid microbubbles were first reconstituted in 2 mL of buffer (0.2 mM HEPES, 2.8 mM NaCl, and 0.05 mM CaCl_2_), and 800 μL of the suspension was then incubated with 10 μg of biotin-labeled Annexin V (Biolegend, San Diego, CA, USA) at ambient temperature for 20 min. Thereafter, the mixtures were washed with phosphate-buffered saline (PBS), and unbound Annexin V molecules were then removed by centrifugation (100× *g*, 2 min). Streptavidin (1 mg mL^−1^; Sigma-Aldrich, St. Louis, MO, USA) in 500 μL of PBS was added to the pellet, and the mixture was incubated at 4 °C for 30 min. After washing the mixture with PBS, 5 μg of biotin-labeled α-human CD56 antibody (Biolegend) was added, and the mixture was incubated at 4 °C for 20 min. Finally, the mixture was washed and resuspended in 300 μL of PBS. To obtain Sonazoid microbubbles–NK cell conjugates (hereinafter NK_Sona cells), 2.5 × 10^6^ NK-92 MI cells were mixed with the labeled Sonazoid mixture at 4 °C for 30 min with continuous rotation at 2 rpm (KRM-5, Korea Biotech, Seongnam-si, Korea).

### 2.3. Viability Assay

The viability of the NK cells immediately after NK_Sona formation was measured using an apoptosis assay kit (Annexin-V-FLUOS Staining Kit, Roche, Mannheim, Germany). Additionally, the viability of the NK_Sona cells after 24 h of formation was detected using the apoptosis assay and the traditional trypan blue exclusion method.

### 2.4. Cytotoxicity Assay

The cytotoxicity of the NK cells and NK_Sona cells was determined according to the method described in a previous study [23]. First, A549 cells were stained with carboxyfluorescein succinimidyl ester (CFSE) using a CellTrace CFSE Cell Proliferation Kit (Invitrogen, Waltham, MA, USA). Then, the stained cells were seeded in 96-well plates at a density of 2 × 10^4^ /well (in 100 μL). Next, NK cells and NK_Sona cells (the latter formed for 24 h) were, respectively, added to the wells of the plates at an effector:target (E:T) ratio of 0.5:1, 1:1, and 2:1. After incubation for 4 h, the cells were harvested and washed twice with PBS. The results were evaluated by DAPI (Promega, Madison, WI, USA) staining and analyzed with a flow cytometer (MACSQuant, Miltenyi Biotec, Bergisch Gladbach, Germany). The percentage of specific cell lysis in each group of cells was determined by measuring the population of double-positive cells in the flow cytometric results.

### 2.5. Degranulation Assay

The activities of the NK and NK_Sona cells were assessed using the CD107a (LAMP-1) degranulation assay. The NK_Sona cells (formed for 24 h) and NK cells were, respectively, seeded in 12-well plates at a density of 5 × 10^5^ cells/well and then activated with 20 ng mL^−1^ phorbol myristate acetate (PMA) and 1 μg mL^−1^ ionomycin (Sigma-Aldrich) for 4 h. Non-activated cells were used as a control. All cells were then stained with CD56-Bright 667/CD107a-Vio515 (Miltenyi Biotec, Bergisch Gladbach, Germany) and analyzed by flow cytometry.

### 2.6. In Vitro Ultrasound Imaging in an Agarose Phantom Model

The echogenicity of the NK_Sona cells was determined using the agarose phantom model with detection on an Affiniti 50 diagnostic ultrasound system (Philips, Amsterdam, The Netherlands). The phantoms were constructed using 2.5% agarose (Sigma-Aldrich) and contained a slanted wall-less channel of 1 mm in diameter. In brief, 2.0 × 10^6^ NK cells or NK_Sona cells were injected into the tubule using a 1 mL syringe, and a 5–12 MHz L12-5 linear transducer was concurrently placed on the phantom to measure the ultrasound signals.

### 2.7. In Vivo Experiments

For the tumor implantation experiments, 6-week-old male BALB/c nude mice (Orient Bio Inc., Seongnam-si, Korea) were subcutaneously injected with 1 × 10^7^ A549 cells and then randomly divided into three groups (*n* = 3 per group). After 3 weeks, 2.0 × 10^6^ NK cells, 2.0 × 10^6^ NK_Sona cells, and PBS were injected intratumorally into the mice three times at 3-day intervals. The tumor size and body weight of the mice were recorded every 2 days after treatment. After 16 days, the mice were sacrificed. All animal experiments were conducted in accordance with institutional guidelines and were approved by the Institutional Animal Care Committee of Chonnam National University (CNU IACUC-YB-R-2020-90, approval date: 12 October 2020).

### 2.8. In Vivo Ultrasound Imaging of Mouse Models of Subcutaneous Tumors

Six-week-old male nude mice were subcutaneously injected with 1 × 10^7^ A549 cells. After 4 weeks, when the tumor size had reached 300 mm^3^, 2.0 × 10^6^ NK cells and 2.0 × 10^6^ NK_Sona cells were intratumorally injected into mice from the respective experimental groups. The mice were then imaged using a 10 MHz linear transducer connected to a 12L3 Siemens ultrasound machine (Vomark Technologies, Wheeling, IL, USA).

## 3. Results

### 3.1. Ultrasound of the Sonazoid-Conjugated Natural Killer Cells

**Figure 1:**
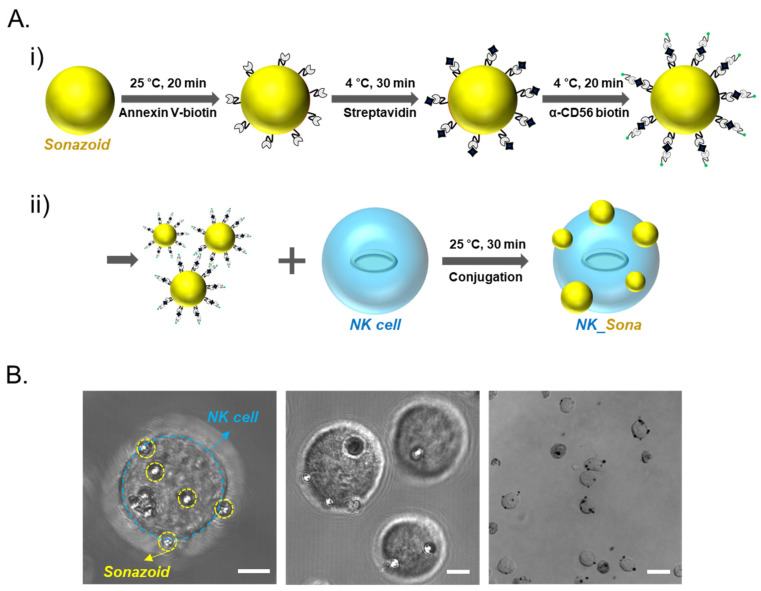
NK_Sona concept and confocal microscopy images. (**A**) Schematic design of the NK_Sona cell formation strategy and biotin/streptavidin-based conjugation. (**i**) Process for the labeling of Sonazoid for binding with the CD56 receptor. Phosphatidylserine lipids on Sonazoid were reacted with Annexin V-biotin, and the intermediates were then conjugated to streptavidin. Finally, the streptavidin-coated microbubbles were conjugated with the biotin anti-human α-CD56 antibody. (**ii**) NK cell membranes were conjugated with the Sonazoid microbubbles via the CD56 receptors at ambient temperature (RT). (**B**) Confocal images of NK_Sona cells after conjugation. The scale bars represent 5 μm, 5 μm, and 20 μm, respectively.

Sonazoid microbubbles were attached to the NK cell membrane through biotin/streptavidin conjugation. In brief, the phosphatidylserine lipids of the Sonazoid microbubbles were sequentially conjugated first to Annexin V-biotin and then to streptavidin, and the resultant conjugate was then coupled with biotin anti-α-CD56 antibody (Figure 1A, top panel i). The CD56 (Neural cell adhesion molecule (NCAM)) receptor, which is related to the activation of NK cells, is a representative marker for NK cells and is generally expressed on surfaces of immune cells inclusive of NK cells [24]. The engineered Sonazoid microbubbles were then linked to the CD56-expressing NK cells (Figure 1A, bottom panel ii). Using optical microscopy, we verified that the labeled microbubbles were capable of binding with the CD56 receptor on the NK cell membrane and had successfully formed complexes with the NK cells (Figure 1B).

**Figure 2:**
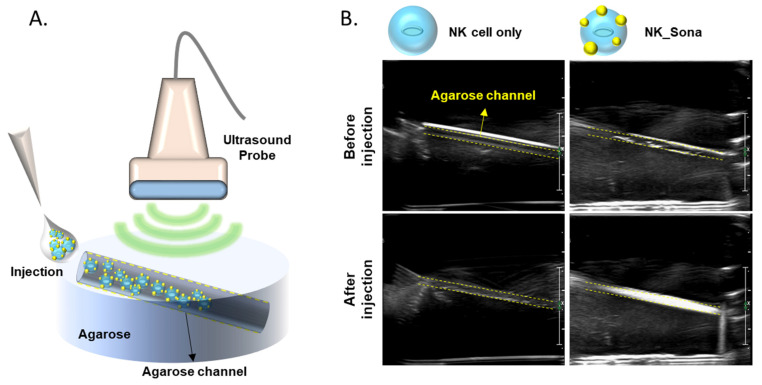
Ultrasound images of NK cells and NK_Sona cells in an agarose phantom model. (**A**) Schematic diagram of the agarose flow phantom model and ultrasound probe. The wall-less tube was 1 mm in diameter and slanted at an angle of 11.31º. (**B**) Ultrasound images of the phantom models before and after injection of the NK cells and NK_Sona cells.

Next, we determined whether the NK_Sona cells could be visualized using an ultrasonic transducer. To this end, the NK_Sona cells were imaged using an agarose phantom model before initiation of the in vivo experiments (Figure 2B). NK or NK_Sona cells were injected into the upper hole of the wall-less straight tube of the phantom model, and the transducer was placed on the model at the same time. As shown in the sonograms in Figure 2B, a clear signal appeared in the tube after the injection of NK_Sona-containing PBS into the phantom model, whereas there was no signal in the tube upon injection of the NK cells.

### 3.2. Viability and Cytotoxicity of NK_Sona Cells In Vitro

**Figure 3:**
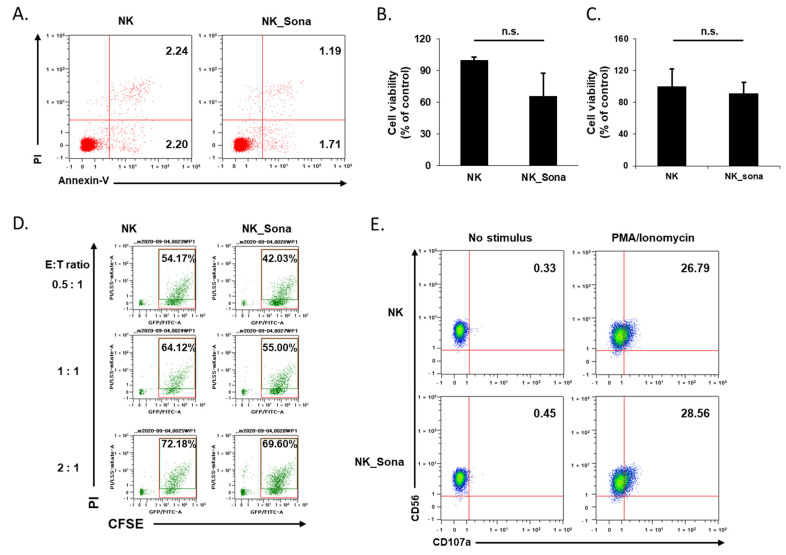
Effects of Sonazoid microbubbles on the viability of NK cells and their cytotoxicity against A549 cancer cells. (**A**) Representative dot plots of FITC-Annexin V/PI staining of NK cells and NK_Sona cells. The apoptosis assay was conducted immediately after NK cell–microbubble conjugation. (**B**) After 24 h, the apoptotic activity of the NK and NK_Sona cells was analyzed using the apoptosis assay with Annexin V/PI staining. (**C**) The viability of the NK and NK_Sona cells was evaluated using the trypan blue exclusion method. (**D**) The cytotoxic activity of the NK and NK_Sona cells was assessed by their specific lysis of A549 cancer cells using the flow cytometry-based cytotoxicity assay. Results are presented as dot plots. NK cells or NK_Sona cells were co-cultured with CFSE-stained A549 cells for 4 h at effector:target (E:T) ratios of 0.5:1, 1:1, and 2:1. (**E**) Representative dot plots of CD107a-specific antibody expression on the NK and NK_Sona cells, with or without PMA/ionomycin stimulation.

Before evaluating the therapeutic effect of the NK_Sona cells, we investigated their viability and cytotoxicity as well as their stability against physical shock during bubble disruption. According to the literature, the physicochemical properties of Sonazoid do not change significantly over 2 h at ambient temperature [22]. Using confocal microscopy, we confirmed that the Sonazoid bubbles on the NK cells had completely collapsed after 24 h incubation at 37 °C (Supplementary material: Figures S1 and S2). Next, the viability of the NK_Sona cells was compared with that of control NK cells using the apoptosis assay and flow cytometric analysis, with the assay conducted at 0 and 24 h after NK_Sona formation. There was no significant difference between the ratios of apoptotic NK cells and NK_Sona cells at 0 h (Figure 3A).

After 24 h incubation, the viability of the cells was analyzed using both the apoptosis assay and trypan blue exclusion method. In both assays, the number of viable NK_Sona cells was slightly reduced (Figure 3B,C). To verify whether the collapsed Sonazoid microbubbles affected the activity of the NK cells, all subsequent experiments were conducted using NK_Sona cells in which the microbubbles on the cell membrane had decomposed. We then assessed the cytotoxicity of NK_Sona cells against A549 lung cancer cells using a flow cytometry-based cytotoxicity assay. There were no significant differences in cytotoxicity between the control NK cells and NK_Sona cells at different E:T ratios (Figure 3D). In addition, we have confirmed that the NK_Sona cells induced apoptotic lysis of the A549 cells in cytotoxic response without the loss of Sonazoid (Figures S1 and S2). We could also have confirmed that the Sonazoid were almost disrupted after 180 min incubation. To verify these results, we analyzed CD107a expression of NK cells (which is related to the cytotoxic activity) [25], in the presence or absence of PMA/ionomycin stimulation, using flow cytometry. Regardless of the stimulation condition, there was no significant difference in the CD107a expression levels between the NK_Sona and NK cells (Figure 3E). Thus, we concluded that the NK cells in the NK_Sona complex had maintained their viability and cytotoxic functions, despite the Sonazoid microbubbles on the cell membranes having collapsed.

### 3.3. In Vivo NK_Sona Cell Imaging

**Figure 4:**
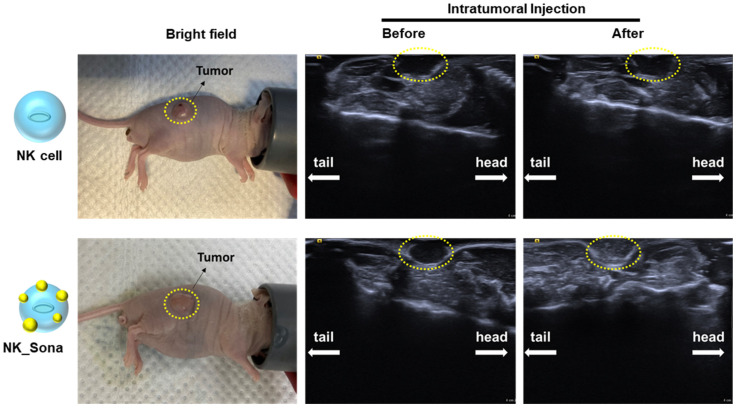
Ultrasound images of NK_Sona cells in mouse models of subcutaneous tumors. NK cells and NK_Sona cells were injected intratumorally into tumor-bearing mice (**left**). The images were taken before (**middle**) and after (**right**) cell injection.

Next, we determined the ultrasound visibility of the NK_Sona cells in vivo. To this end, BALB/c nude mice were injected subcutaneously with A549 lung cancer cells to establish the animal xenograft model. Once the tumors had reached a size of 300 mm^3^, the mice were injected intratumorally with NK cells or NK_Sona cells, and the animals were then subjected to ultrasound imaging to determine whether the therapeutic cells could be visualized. The NK_Sona cells were indeed detected by the ultrasound probe owing to the label-conjugated Sonazoid microbubbles on their membrane surface (Figure 4). By contrast, the control NK cells were not detectable on the sonogram.

### 3.4. Therapeutic Effect of the NK_Sona Cells In Vivo

**Figure 5:**
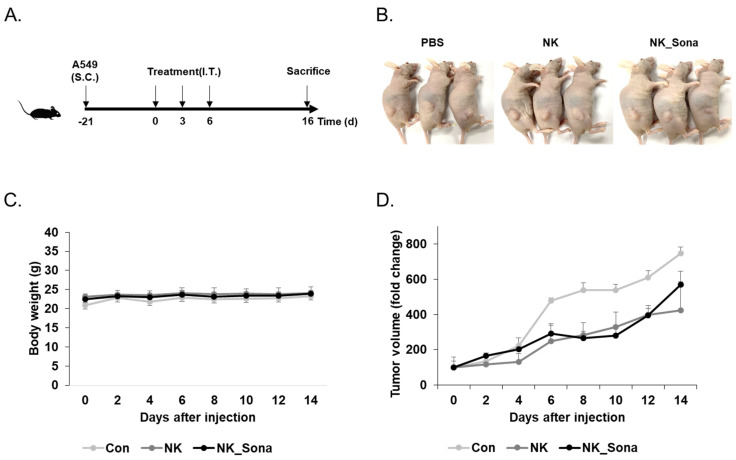
Therapeutic effect of NK_Sona on a mouse model of subcutaneous tumors. (**A**) Schematic diagram of the *in vivo* experimental schedule. (**B**) Images of tumor-bearing mice treated with control NK cells, NK_Sona cells, or PBS (control). All images were taken on day 16 after treatment. The body weights (**C**) and tumor volumes (**D**) were measured every 2 days for 16 days. The tumor volumes were normalized to those measured on day 0. Data are represented as the mean S.D. (*n* = 3).

Finally, the therapeutic effect of the NK_Sona cells was evaluated using tumor-bearing mice. At 3 weeks after subcutaneous A549 injection, the tumor-bearing mice were administered the NK cells, NK_Sona cells, or PBS (as a control) and were then assessed for tumor growth, which was measured at 2-day intervals for 16 days. The schematic design of the in vivo therapeutic experiment is provided in Figure 5A. There were no significant differences in body weight among the mice of all three groups (Figure 5B). Additionally, the NK_Sona cells inhibited tumor growth as much as the control NK cells did during the experimental period (Figure 5C,D). In summary, the NK_Sona cells could be visualized in real time and were not functionally affected by the Sonazoid microbubbles on their cell membranes.

## 4. Discussion

Many cell therapy strategies for solid tumors rely on enhanced cell functions, such as CAR-expressing T/NK cells [26,27]. Although such therapies have improved the clinical treatment of liquid cancers, that of solid tumors remains limited [10,28]. Thus, we considered the use of microbubbles as a new approach for the physically based infiltration of NK cells into tumor cells. As a starting point, we assessed the feasibility of using Sonazoid microbubbles conjugated to NK cells as a new method for the ultrasound imaging of therapeutic cells and then treated solid tumors with the engineered NK_Sona cells. Subsequently, we verified by microscopy observation that the Sonazoid microbubbles were successfully attached to the NK cell membrane. Additionally, we created a simple phantom model to assess the visibility of the NK_Sona cells and confirmed that the engineered cells could indeed be detected using an ultrasound system. Furthermore, the conjugation step did not affect the viability of the NK_Sona cells, although their number and viability were slightly decreased after 24 h. Interestingly, the cytotoxic function of the NK_Sona cells was maintained after 24 h of incubation, indicating that they could effectively be used for cancer cell treatment. Finally, our in vivo study confirmed that the NK_Sona cells could be clearly detected by ultrasonography in tumor-bearing mice. Taken together, our findings verified that it was possible to visualize NK cells in real time via their conjugation with Sonazoid without any decline in their therapeutic effect. Thus, we have demonstrated the feasibility of NK_Sona cell application for real-time imaging and cancer therapy based on the ultrasound system. However, our studies were conducted without control of the NK_Sona-based ultrasound system. This system is currently under further development for allowing the simultaneous real-time visualization and control of the contrast agent. Thus, in the future, we will further investigate the efficacy of NK_Sona cell infiltration into solid tumors based on ultrasound focusing. In conclusion, our new approach using NK_Sona cells can be a promising strategy for overcoming the limitations of solid tumor treatment using cell therapy.

## 5. Conclusions

We developed Sonazoid microbubble-conjugated natural killer cells (NK_Sona) and performed a viability, cytotoxicity, and degranulation assay to see the effect of the NK_Sona on the NK cells. Moreover, we successfully demonstrated real-time ultrasound imaging and showed the therapeutic effect via in vivo experiments using BALB/c nude mice. Further development of the ultrasound system is underway for realizing the eventual clinical application of these engineered NK_Sona cells.

## Data Availability

Data are available upon request.

## Supplementary Materials

The following are available online at https://www.mdpi.com/article/10.3390/pharmaceutics13101689/s1, Figure S1: Confocal microscopy images of NK_Sona and A549, Figure S2: Confocal time series in cytotoxic response.
