# Supplementary material for: Sonazoid-Conjugated Natural Killer Cells for Tumor Therapy and Real-Time Visualization by Ultrasound Imaging
Source: Pharmaceutics. 2021 Oct 15;13(10):1689. doi: 10.3390/pharmaceutics13101689 (PMC8537855; doi:10.3390/pharmaceutics13101689)
Supplement: Supplementary file 1 [file pharmaceutics-13-01689-s001.zip › pharmaceutics-1352523-supplementary.pdf]

pharmaceutics

MDPI

Supplementary Materials: Sonazoid-Conjugated Natural Killer Cells for Tumor Therapy and Real-Time Visualization by Ultrasound Imaging

Hyeong-Woo Song, Han-Sol Lee, Seok-Jae Kim, Ho Yong Kim, You Hee Choi, Byungjeon Kang, Chang-Sei Kim, Jong-Oh Park and Eunpyo Choi

To distinguish and label apoptotic cells, we used CellEvent Caspase-3/7 green detection reagent. As shown in Figure S1, we have verified the NK_Sona cells, which Sonazoid were shown black or white dots, nearby green stained A549 cells. Additionally, to verify the distribution of the Sonazoid in cytotoxic response, we have confirmed that NK_Sona cells interact with A549 cells by real-time confocal microscopy. Figure S2 shows the Sonazoid (black or white dots in the bright field) on the NK cells membrane, which were induced apoptotic lysis of A549 cells. After 180 min of incubation at microscopy, most Sonazoid was invisible because it was destroyed naturally. Therefore, these results indicated that the NK_Sona cells are induced killing of the tumor cells, and Sonazoids are not lost during the killing process.

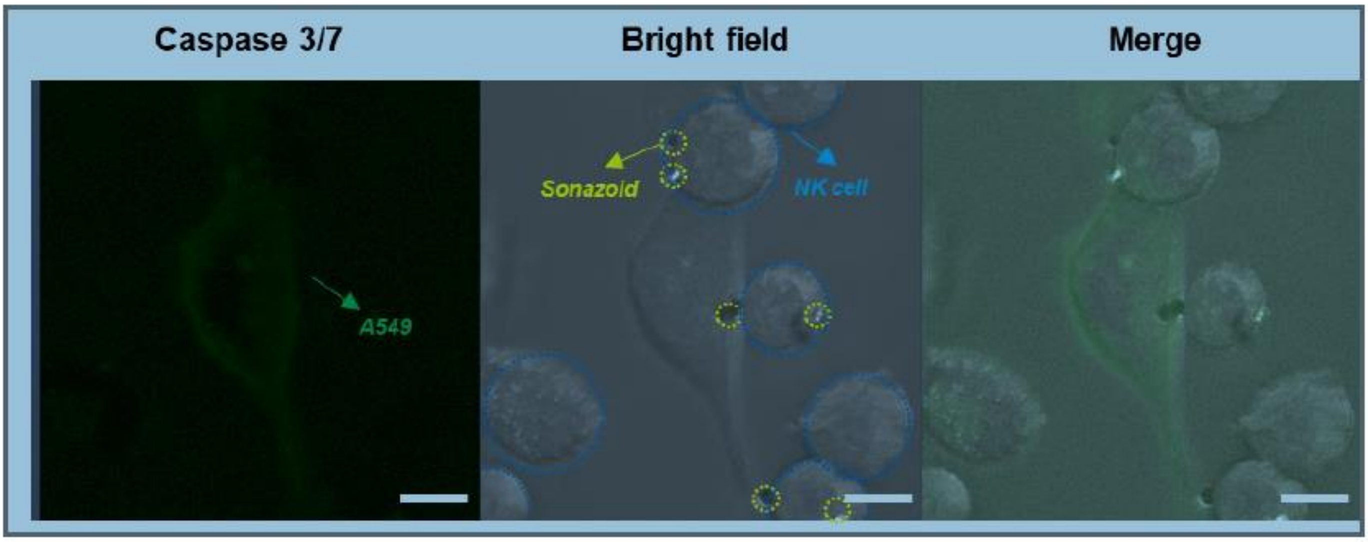

Figure S1. Confocal microscopy images of NK_Sona and A549. The scale bars represent 10 µm.

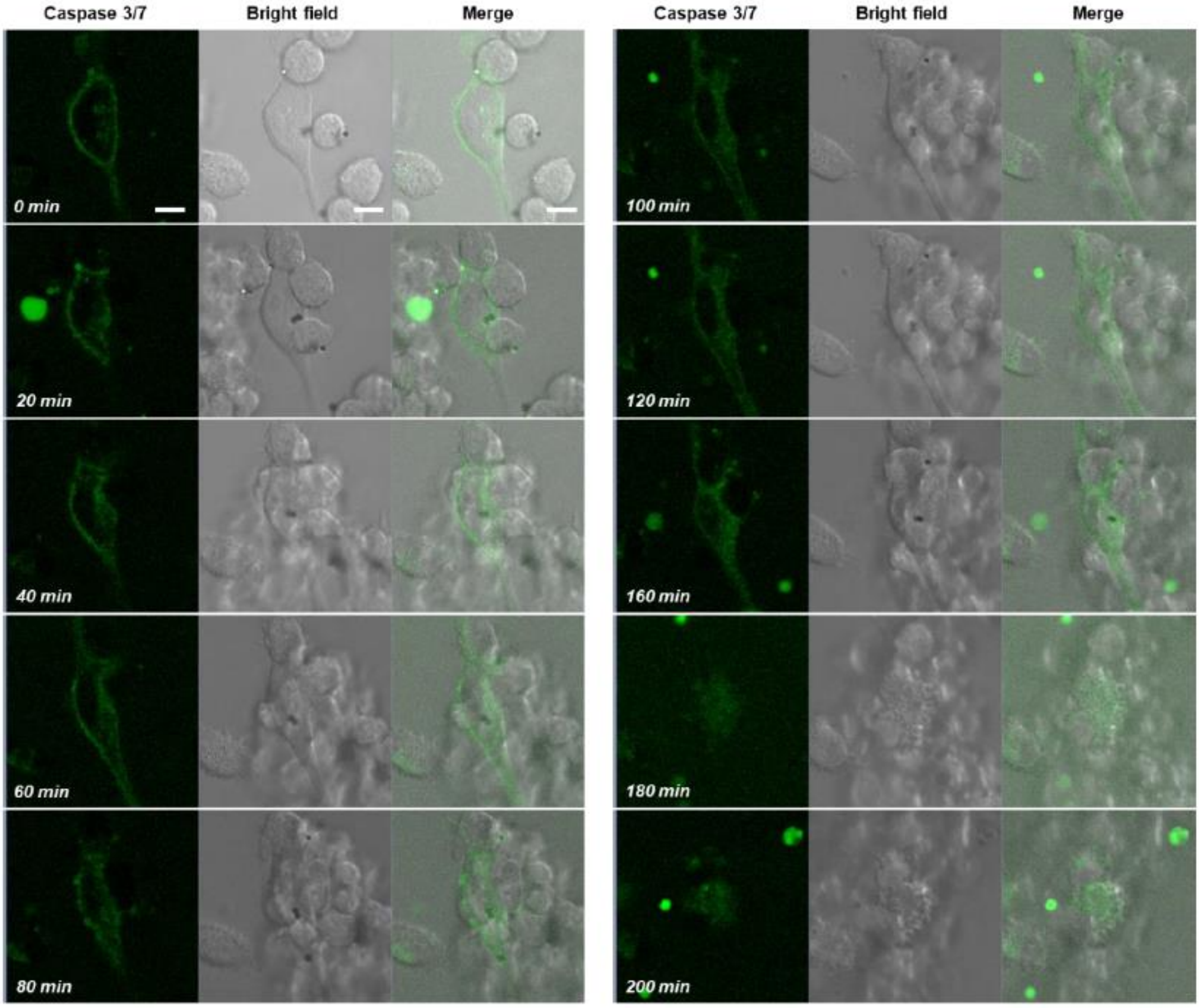

Figure S2. Confocal time series in cytotoxic response. The scale bars represent 10 μm.
